# Supplementary material for: Theoretical and Experimental Study of Different Side Chains on 3,4-Ethylenedioxythiophene and Diketopyrrolopyrrole-Derived Polymers: Towards Organic Transistors
Source: Int J Mol Sci. 2024 Jan 16;25(2):1099. doi: 10.3390/ijms25021099 (PMC10816275; doi:10.3390/ijms25021099)
Supplement: Supplementary file 1 [file ijms-25-01099-s001.zip › ijms-2804743-supplementary.pdf]

Supplementary Materials

Figure S1. GPC for the polymers P1 (left) and P2 (right).

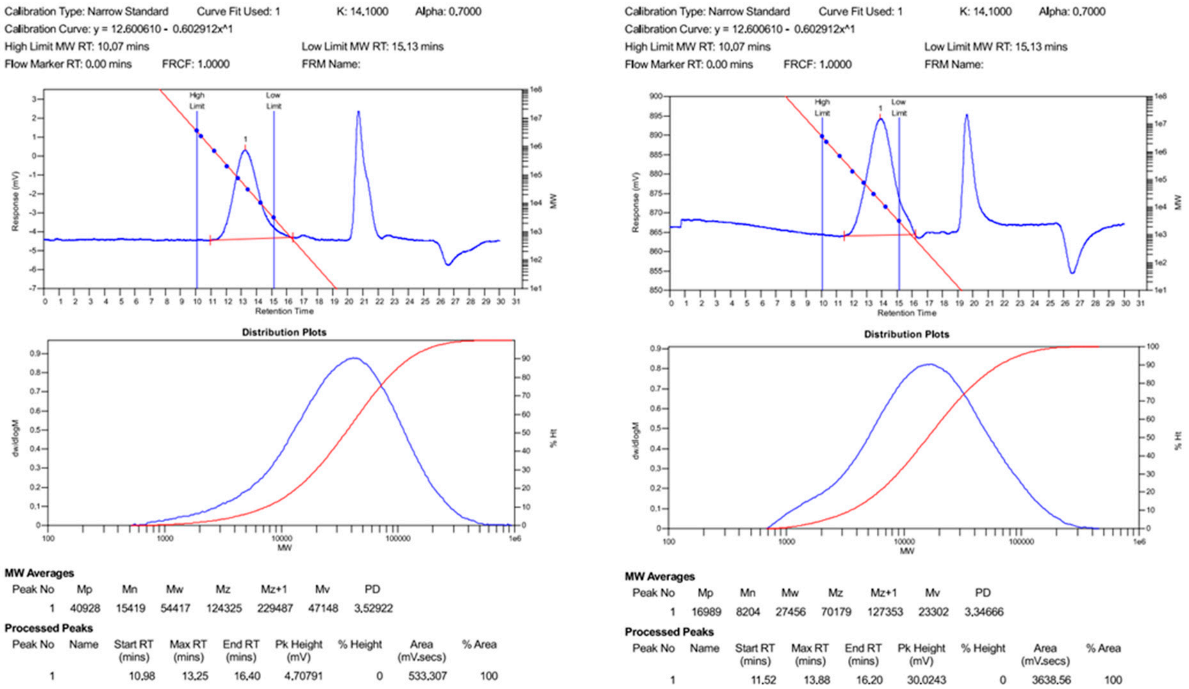

Figure S2. DSC curve of the polymers P1 and P2.

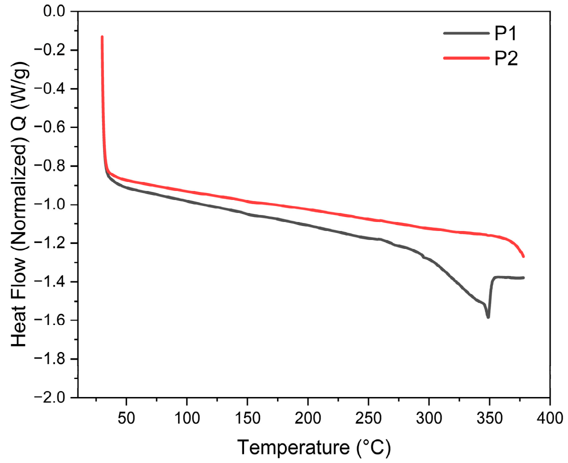

Figure S3. The orbital of HOMO−1, LUMO+1, HOMO−2, and LUMO+2 of the dimer.

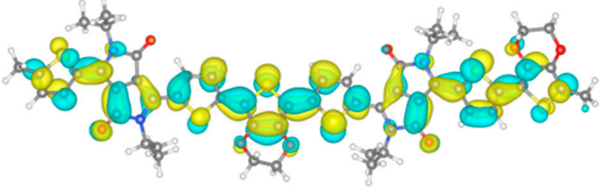

LUMO+2

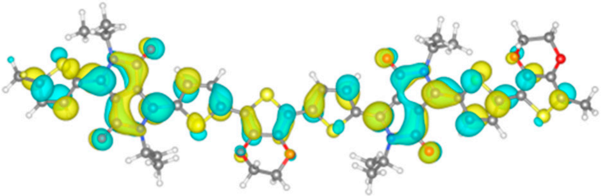

LUMO+1

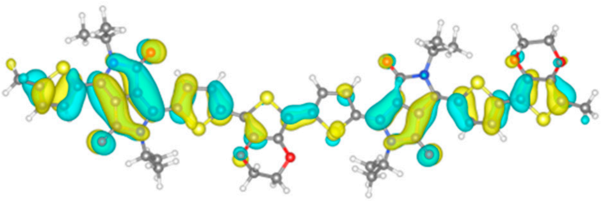

HOMO−1

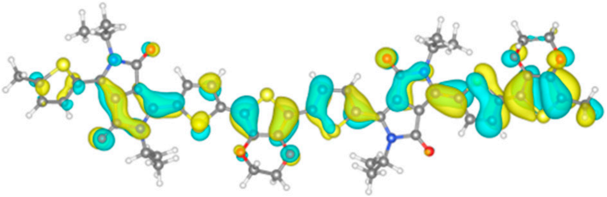

HOMO−2

Figure S4. Theoretically modelled UV absorption characteristic curves for dimer.

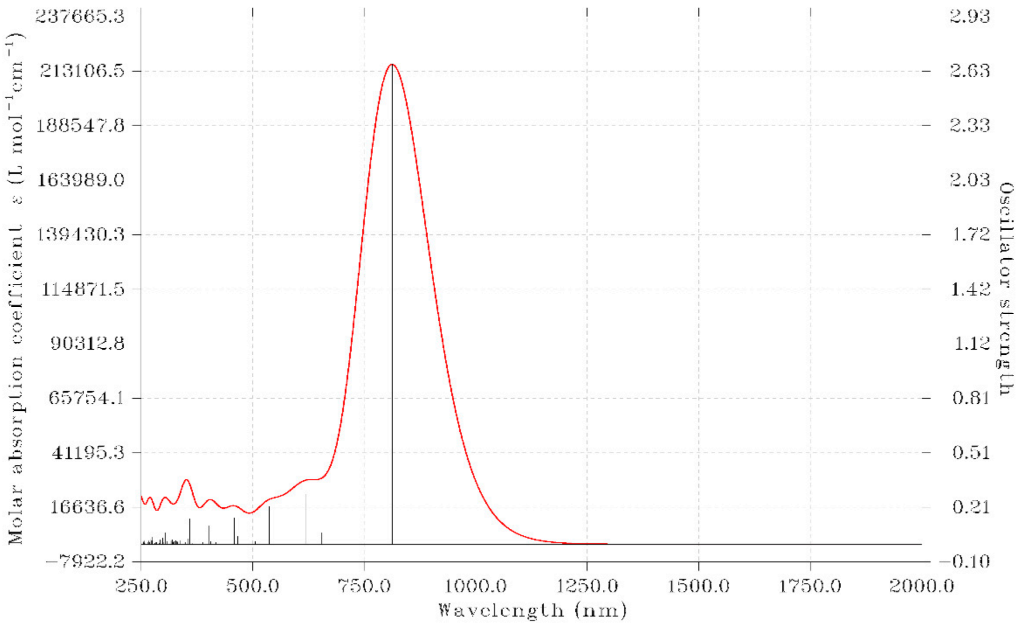

Figure S5. The device configuration of BG/BC OFET.

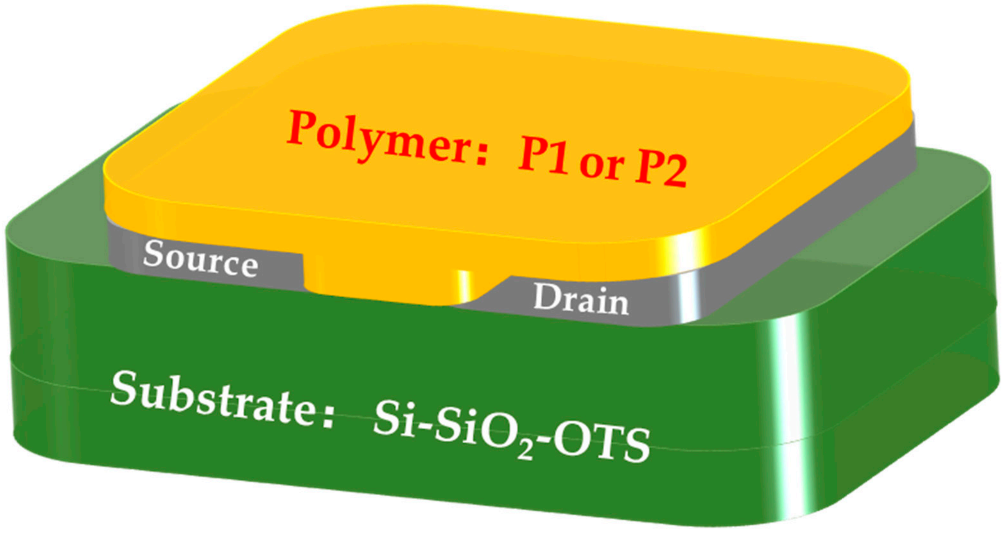

Figure S6. The 3D topography of the polymers P1 (left) and P2 (right).

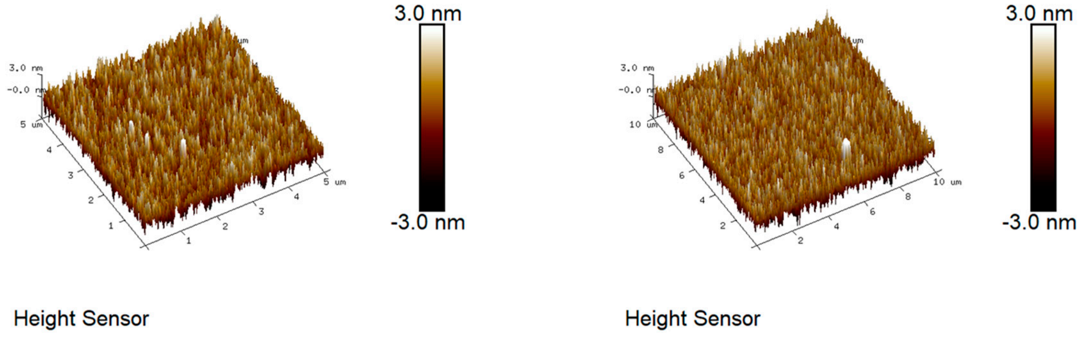

Table S1. Space atomic coordinates of the dimer.

C	-8.73605899	-0.45369890	-0.29507601
C	-7.32904897	-0.39878691	-0.59663301
C	-6.74327594	0.58302408	-1.39939601
C	-5.34485096	0.48918207	-1.48565201
C	-4.81118997	-0.55763293	-0.73655301
S	-6.08292199	-1.43630592	0.08163699
C	-3.43555797	-0.93112194	-0.59084201
S	-2.14711496	-0.03413995	-1.38694801
C	-0.90242597	-1.07454296	-0.70503101
C	-1.48138298	-2.05323296	0.09236899
C	-2.90112098	-1.97246795	0.15692999

O	-0.77971399	-3.00046897	0.74884099
C	-1.59110200	-4.09537896	1.20933299
C	-2.87193799	-3.58589995	1.83815999
O	-3.64254199	-2.83331794	0.88410499
C	0.48613603	-0.87479398	-0.99821801
C	1.06493404	0.10463002	-1.80206801
C	2.46452903	0.01066901	-1.87966901
C	3.00219302	-1.05211699	-1.15058001
S	1.71583102	-1.92566599	-0.33168701
C	4.41387902	-1.29963200	-1.00740001
N	-9.47177698	-1.51736889	0.23843799
C	-10.83334698	-1.12448388	0.48513399
C	-10.89969199	0.25720612	0.05734999
C	-9.61905398	0.62536711	-0.44316201
C	5.41390002	-0.34175601	-1.22245901
C	6.67991802	-0.88993602	-0.87731201
C	6.47237102	-2.26544602	-0.47858801
N	5.05424402	-2.46894901	-0.58444501
C	-11.71539194	1.39027812	0.12807899
N	-10.97949297	2.46094912	-0.38209501
C	-9.65432597	2.04090511	-0.74876301
O	-11.67807600	-1.89704288	0.91914999

O	-8.83661896	2.80307910	-1.25187601
C	-9.16278501	-2.95455290	0.36489399
C	-11.39257992	3.81580012	-0.79454501
C	-13.04857195	1.40281413	0.68584199
C	-13.86267198	0.28140914	0.78710999
C	-15.08114199	0.52108015	1.46977899
C	-15.21672094	1.81664615	1.92060999
S	-13.82135393	2.76272514	1.49147899
O	7.24438601	-3.16287703	-0.15552201
C	4.57087000	-3.85780700	-0.46474001
C	5.60124706	1.06441099	-1.51101601
N	7.00217906	1.30030998	-1.29570901
C	7.64866703	0.12802397	-0.88870701
O	4.83581505	1.93728299	-1.90361201
C	7.52514706	2.61216397	-1.72267901
C	9.02074104	-0.05005304	-0.49547301
S	10.13314303	1.21406995	0.01833099
C	11.39653303	0.05843094	0.36997099
C	10.95863905	-1.23743405	0.10487899
C	9.63694202	-1.29776505	-0.36625101
C	-8.57307998	-3.56057390	-0.90877301
C	-8.41164097	-3.29119290	1.65468999

C	-12.68777496	3.83266313	-1.60585001
C	-11.34026493	4.82142712	0.35676799
C	3.76347000	-4.32308000	-1.67666501
C	3.93085400	-4.15076900	0.89386799
C	8.64698508	2.50819996	-2.75626801
C	7.79914904	3.55588097	-0.54972901
C	12.67393602	0.48134793	0.86762599
C	13.10856403	1.76853192	1.14340898
C	14.45103004	1.82863491	1.63992399
C	15.05460904	0.59715891	1.74753599
S	13.95804104	-0.66703608	1.23783199
O	12.35089207	2.87014693	0.96047499
C	12.88067709	4.04777393	1.59338099
C	14.37976109	4.13820492	1.38885499
O	15.04262207	3.00676591	1.96568499
C	-16.35196492	2.42883616	2.67515099
C	16.43267106	0.30053290	2.23614999
H	-7.33240898	1.36091909	-1.88290801
H	-4.73113395	1.16840707	-2.07914801
H	-1.81460400	-4.76107995	0.35815199
H	-0.99144000	-4.64329296	1.94754199
H	-3.50233100	-4.42423794	2.16147799

H	-2.64931298	-2.94377095	2.70756899
H	0.48297604	0.86966003	-2.31840801
H	3.09276904	0.71081001	-2.42791201
H	-10.16602298	-3.38569889	0.48511999
H	-10.58175692	4.09954211	-1.47947501
H	-13.56085899	-0.68893686	0.39656599
H	-15.84700995	-0.23926385	1.63136399
H	5.51344201	-4.42184801	-0.47634701
H	6.65626608	3.03507398	-2.24477001
H	11.58625904	-2.11718706	0.25403199
H	9.11082504	-2.22567004	-0.58774201
H	-8.58208001	-4.65793190	-0.81844301
H	-9.18426997	-3.28675790	-1.78264101
H	-7.53698301	-3.25381091	-1.10164701
H	-8.34999802	-4.38513491	1.76773499
H	-7.38713299	-2.89679891	1.68052199
H	-8.95361999	-2.88901190	2.52422599
H	-12.64841396	3.08346613	-2.41162301
H	-13.58387894	3.64423814	-1.00062301
H	-12.80336695	4.82455713	-2.06968001
H	-11.52096493	5.83629911	-0.03125201
H	-12.08863390	4.62356013	1.13598899

H	-10.34534695	4.80838511	0.82735999
H	4.30192500	-4.09165600	-2.60869801
H	3.63492099	-5.41526498	-1.62089601
H	2.76295700	-3.87542199	-1.73497401
H	3.73292999	-5.23091400	0.98041599
H	4.61724300	-3.86430300	1.70526699
H	2.97960501	-3.62520099	1.05165499
H	8.35006607	1.83595796	-3.57614701
H	8.83055607	3.50586096	-3.18468101
H	9.59640107	2.14730195	-2.34043301
H	6.90379909	3.63821598	0.08534399
H	8.63693907	3.23753296	0.08454299
H	8.03647709	4.55980897	-0.93585001
H	12.37307205	4.90632693	1.13455999
H	12.63861508	4.01783093	2.66988999
H	14.61561109	4.19656991	0.31098599
H	14.77803806	5.03118291	1.88896499
H	-17.13266096	1.67427116	2.84853099
H	-16.02707996	2.81897116	3.65412899
H	-16.80223594	3.26963516	2.12154399
H	17.01005505	1.23456990	2.29499799
H	16.42204502	-0.15600510	3.24115799

H	16.96322805	-0.39371010	1.56486899
